# Influence of Turn-Taking in Musical and Spoken Activities on Empathy and Self-Esteem of Socially Vulnerable Young Teenagers

**DOI:** 10.3389/fpsyg.2021.801574

**Published:** 2022-02-07

**Authors:** Sarah Hawkins, Camilla Farrant

**Affiliations:** ^1^Centre for Music and Science, Faculty of Music, University of Cambridge, Cambridge, United Kingdom; ^2^Music Therapy Tree, London, United Kingdom

**Keywords:** turn-taking, improvisation, music, language, empathy, self-esteem, socially-vulnerable, rhythm

## Abstract

This study describes a preliminary test of the hypothesis that, when people engage in musical and linguistic activities designed to enhance the interactive, turn-taking properties of typical conversation, they benefit in ways that enhance empathy and self-esteem, relative to people who experience activities that are similar except that synchronous action is emphasized, with no interactional turn-taking. Twenty-two 12–14 year olds identified as socially vulnerable (e.g., for anxiety) received six enjoyable 1-h sessions of musical improvisation, language games that developed sensitivity to linguistic rhythm and melody, and cross-over activities like rap. The Turn-taking group (*n* = 11), practiced characteristics of conversation in language games, and these were also introduced into musical activities. This involved much turn-taking and predicting what others would do. A matched control group, the Synchrony group, did similar activities but in synchrony, with less prediction and no turn-taking. Task complexity increased over the six sessions. Psychometric testing before and after the series showed that the Turn-taking group increased in empathy on self-report (Toronto Empathy Questionnaire) and behavioral (‘Reading the Mind in the Eyes’) measures, and in the General subtest of the Culture-Free Self-Esteem Inventory. While more work is needed to confirm the conclusions for relevant demographic groups, the current results point to the social value of musical and linguistic activities that mimic entrained, tightly coordinated parameters of everyday conversational interaction, in which, at any one time, individuals act as equal participants who have different roles.

## Introduction

There is good evidence that any sort of shared rhythmicity (e.g., joint music-making, hand-clapping games, football chants) enhances self-esteem and a sense of group identity, see e.g., Miles et al. (2009) and references therein. These joint actions usually make people feel happier, experience other people as happier and friendlier, and encourage them to behave in a more prosocial or empathetic way, independent of language communication skills *per se* (Warner et al., 1987; Behrends et al., 2012; Rabinowitch et al., 2013; Cirelli et al., 2016) though not of initial propensity for cooperation (Lumsden et al., 2012). In a series of experiments, Trainor, Cirelli and colleagues demonstrated not only that prosocial behavior is induced by shared rhythmic engagement in the first few years of life, but also that prosociality generalises to other people who behave as a member of the social group, and not to people who behave as if they do not belong (Cirelli et al., 2014, 2016; Trainor and Cirelli, 2015). Physiological stress indicators also tend to decrease during choral singing and during group drumming, both of which demand shared rhythmicity (Fancourt et al., 2016b,c). Broadly, social and health benefits from group music-making seem to stem from joint attention to the shared music, probably particularly the beat, or pulse, and do not require developed language.

Joint rhythmic actions require temporal entrainment, manifest in group music-making as a shared pulse. Neuroscientific research confirms that a shared musical pulse induces temporal entrainment between ensemble players (Lindenberger et al., 2009; Sänger et al., 2012). Likewise, convergent literature suggests that successful spoken communication includes temporal entrainment (Wilson and Wilson, 2005; Hasson et al., 2012; Pérez et al., 2017). However, while some forms of talking can be very rhythmic—notably poetry and parts of rhetorical speech—conversation is the most common but the least rhythmic style of speech. It is usually said to exhibit no extended rhythmicity at all (cf. Knight, 2014). Yet paradoxically, most people find conversation rewarding in itself. While non-rhythmic aspects of conversation (e.g., its content) presumably provide rewards, the high degree of coordinated behaviors, including gesture, exhibited between interacting talkers suggests that rhythmic entrainment between interlocutors is likely to arise, if only at times. When entrainment arises, it should further reward interactants. So how and where do shared pulse and entrainment typically develop in conversation?

One recent line of phonetic research indicates that shared pulse may be very important in conversation in that it seems to indicate positive social alignment at turn transitions (Hawkins et al., 2013; Hawkins, 2014; Ogden and Hawkins, 2015). The terms turn and turn transition come from Conversation Analysis (Schegloff, 1991, 2007). Broadly, a turn is when one person in a conversation principally holds the floor, though other people may contribute during the turn too, as when they interject ‘uh-huh,’ or nod during the conversation. A turn transition occurs when the current talker gives up the floor and another person becomes the principal talker. Particular, complex factors govern how the end of one turn is signaled and the next turn begins. The details differ depending not so much on what is being talked about, but on the function [the purpose(s)] of this part of the conversation. For example, whether the first talker expects, and the second talker signals, agreement or disagreement, whether the second talker seeks to continue the topic or to change the subject, and so on. These details include choice of words and grammatical structure, and both talkers’ use of a host of phonetic resources, many of which are musical: relative pitch, timing, voice timbre and articulatory properties that correspond broadly with musical attack (Local, 2003; Local and Walker, 2005; Zellers and Ogden, 2014). Ogden and Hawkins (2015) showed that, in Question-Answer pairs, the person asking the question uses stressed (accented) syllables to set up a brief regular pulse at the end of the question, and when the answer is ‘well-formed’ with respect to the question, it typically begins with that same pulse. In contrast, when the answer is not well-formed for whatever reason (it is not a ‘preferred’ response), it typically begins off-beat. We conjectured that aligning rhythmicity in these ‘local’ places may be enough to maintain successful communication and to gain the personal and social benefits of temporal entrainment that arise from shared pulse. That is, mutual entrainment may be essential to successful conversation only at turn transitions, yet still enhance well-being.

In related work, Hawkins et al. (2013) showed that when pairs of individuals talk while they are improvising music, the talk and the music share a more similar pulse when the music is judged to be more successful. Furthermore, changes in pulse transfer seamlessly between individuals and between domains (music or speech). Thus parameters governing the establishment of interpersonal alignment seem to be essentially the same for music and speech, regardless of musicianship (see also Hawkins, 2014; Robledo et al., 2016). This finding suggests that benefits gained by interactive work in music might transfer to language interaction.

In musical performance, there are many parallels with conversational turns and turn transitions. For example, call and response songs, but in these, each turn may last much longer than the average conversational turn, and the exchange of turns is formalized so that prediction is easy. A closer parallel with conversation is when a solo line is improvised together with an improvised accompaniment that provides additional, brief, musical interest between phrases. Such structures are inherent in musical genres like gospel singing and jazz ensembles. They were discussed for example by Monson (1996). The leader–follower relationship in musical improvisation has parallels with partners in spoken conversation, and people are sensitive to coordinated body movement between players (e.g., Moran et al., 2015). Interestingly, though it is generally accepted that temporally coordinated communicative body movement is used by all competent musicians, it is more prevalent during non-pulsed (free) than for pulsed jazz improvisation, presumably to compensate for the lack of an external pulse (Eerola et al., 2018). Doffman (2011) notes similar effects for eye gaze and how the player moves their instrument at the end of an improvised song, when coordination between players is particularly critical and challenging.

The music therapy community also typically accepts that turn-taking in musical interaction leads to enhanced social behaviors since it mirrors early mother-infant interaction (Sutton, 2002; Holck, 2004). No systematic study in music therapy literature specifically connects musical turn-taking with empathy or self-esteem. However, case studies and some research studies suggest that music therapy in general improves conversational turn taking for children with social and verbal impairments (Edgerton, 1994; Aldridge et al., 1995; Oldfield, 1995; Holck, 2004).

Pulling these literatures and observations together, we reasoned as follows. Joint music-making is typically rewarding and enhances group affiliation, presumably at least partly through shared rhythmicity—shared pulse. One could expect this experience to generalize to shared rhythmicity in language, especially since people typically report finding pleasant conversation rewarding, but the problem with this is that there is no evidence for a sustained steady pulse in ordinary conversation. However, since there is evidence for rhythmicity being established at the end of one talker’s turn, and maintained across the turn by the next talker, albeit briefly, this might provide not just sufficient reward for the sense of affiliation to develop, but a more effective type of affiliative action (or reward) than simply maintaining joint rhythmic action without a need for significant interaction. Support for this speculation comes from the well-established behaviorist observation that partial (intermittent and random) positive reinforcement facilitates longer-lasting learning than regular reinforcement. Venturing beyond behaviorist terms, we could conceptualize this as engendering hope. Moreover, entrainment at turn transitions ensures that each interactant attends closely to the other’s behavior and tries to predict when their own turn will start. Such close attention to others helps choral, synchronous rhythmic action, but is by no means essential to it as long as the external pulse itself is predictable. Furthermore, such turn-taking interaction, with quasi-independent yet tightly coordinated roles, could potentially offer richer sources of information about one’s interactant(s) than does playing or singing in synchrony with a shared pulse. We therefore asked whether shared rhythmicity is most beneficial to social interaction when joint activities focus attention onto rhythm across interactive turns. Specifically, we predicted that positive changes in empathy and self-esteem will be greater when activities are targeted to enhance joint interpersonal attention to shared rhythm at turn transitions, rather than to enhance general synchronicity of rhythm across longer stretches of behavior without need for interaction.

Since temporal entrainment is a general process experienced by the vast majority of people, then if the hypothesis is right, everyone should benefit from attention to shared rhythmicity at turn transitions. We chose to test this hypothesis with moderately socially-vulnerable people, for three reasons. First, as a service: this type of population could benefit from enhanced empathy and self-esteem as well as better communication skills. Second, to avoid ceiling and floor effects: most people in socially-vulnerable populations are likely to have moderately low empathy and self-esteem. Assuming an ogival function in the rate at which change can be achieved, to start from moderate scores should maximize the chances of inducing measurable change from short-term intervention, compared with people who score at either extreme on these measures. Thirdly, for this proof-of-concept study we wanted to avoid disabilities that would make execution of the tasks difficult: people who have physical or learning challenges might take longer, or need more individualized methods, to benefit.

The final program was carried out with young teenagers who were identified as having emotional problems that affected them both socially and academically. We chose this demographic group since they fulfilled the three criteria above and their schools’ inclusion units could accommodate the program.

We measured empathy rather than prosocial behavior because empathy can be assumed to lead to prosocial behavior, and can be measured reliably via validated questionnaire, whereas it proved impractical to objectively or subjectively measure prosocial behavior of young teenagers in their schools. Self-esteem was likewise assessed by questionnaire.

In sum, this study developed and delivered a program of enjoyable musical and verbal interactive activities (games) that stress shared rhythm, and are intended to enhance empathy, self-esteem and prosocial behavior, including more effective interactive communication. We delivered the program in two contrasting ways to two groups of participants. A Turn-taking group spent significant time learning to ‘pass turns’ fluently between them, modeling properties of conversational interaction, translated as needed into music. A parallel Synchrony group did most of the same musical and language tasks, but always in synchrony, typical of much Western group music-making. We predicted that positive changes in empathy and self-esteem would be greater for the Turn-taking group, whose games were targeted to enhance joint attention to shared rhythm across interactive turns (i.e., at turn transitions), rather than to enhance attention to general synchronicity of rhythm. The hypothesis is novel, and the training requires several weeks’ commitment from participants, so this first study is necessarily exploratory. Its promising results are reported in full to allow further investigation.

## Materials and Methods

### Participants

Twenty-four children were recruited by the Special Educational Needs Coordinators (SENCOs) at two Cambridgeshire (United Kingdom) state schools, of whom 22 completed the course. The children who dropped out, one boy and one girl, were from the same school. The boy was asked to leave because his behavior prevented the group from doing any activity, and the girl’s parents preferred her to attend another class scheduled at the same time. All children attended the ‘inclusion’ unit of their school. They were selected by SENCOs as suffering from low self-esteem and/or empathy, and related problems such as anxiety, and as likely to enjoy the sessions offered in that they liked music. Because of the emphasis on interpersonal interaction, no child had a diagnosis of autism or Asperger syndrome, or presented primarily with autistic traits. Mean ages at time of first testing (see next Sections) were as follows. Girls: 12.89 years, range 12.14–14.13; boys 13.08, range 12.24–14.18. Mean age of the Turn-taking group (five girls, six boys) was 12.84 years, range 12.26–14.18, and of the Synchrony group 13.12 years, range 12.14–14.13.

Fully informed parental consent was obtained, and each child also signed consent forms before the first session, and at the end of the last session. Information provided, and the forms themselves, were approved by the Ethics Committee of the School of Humanities and Social Sciences, University of Cambridge. Each child who completed the course received a £25 gift card in the last session and received a certificate from the University of Cambridge Music Faculty.

### Design

We used a fully counterbalanced 2 × 2 between-groups design with Treatment Group (Turn-taking or Synchrony) and Sex (Female, Male) as independent variables. Dependent variables were scores on psychometric tests, administered once before sessions began, and once afterward.

Table 1 shows how participants were assigned to conditions. Children were divided into four subgroups of (initially) six participants. Each subgroup was single sex, and each school had one group of boys and one of girls. Within each school, one group was assigned to the Turn-taking condition and the other to the Synchrony condition. Thus Treatment Group sizes were equal, and sex counterbalanced across treatment conditions within schools.

**TABLE 1:** Number of participants in each Group (Turn-taking, Synchrony) according to their sex and school.

	Treatment Group				
	Turn-taking		Synchrony		Total
School	School1	School2	School1	School2	
Female	5	–	–	6	11
Male	–	6	5	–	11
Total	11		11		22

Since the boy and girl who dropped out were from the same school, there were still equal total numbers (11) in each Treatment group: five girls and six boys in Turn-taking, and six girls and five boys in Synchrony. While group sizes of 11 risk low statistical power, access to near-clinical populations such as these is difficult, and working with them is challenging. For a first test of concept, accepting Ns of this size was a practical decision dictated by budget and access.

### Psychometric Tests

Psychometric testing was carried out before and after the series of music and language sessions. There were two self-report questionnaires and one behavioral test, chosen in consultation with the funding body and the schools. The self-report tests were the Toronto Empathy Questionnaire^1^ (TEQ, Spreng et al., 2009) and version 3 of the Culture-Free Self-Esteem Inventory^2^ (CFSEI-3, Battle, 2002). The behavioral test was Reading the Mind in the Eyes Test^3^ (RMIE, children’s version, Baron-Cohen et al., 2001b), since renamed the Eyes Test. The TEQ was normed on 18-year-old undergraduates, but School1 preferred it to the more age-appropriate Bryant Index of Empathy (Bryant, 1982), because the children were familiar with TEQ’s 5-point response scale, and not the Bryant’s binary yes/no measure. The CFSEI-3 was chosen because the funding body uses it and wanted comparability with their own measures. The RMEI offered a simply administered objective measure of behavioral change. Sections “Empathy,” “Self-Esteem (Version 3 of the Culture-Free Self-Esteem Inventory),” and Supplementary Materials provide more information about test content.

### Instruments and Other Apparatus

Musical instruments were 12-note pentatonic balaphons (marimbas), djembe drums, and tambourines. These were chosen from experience in related work (Hawkins et al., 2013) and piloting for this work. Pentatonic melodic instruments were chosen because we wanted harmonious sounds, rarely achieved when non-musicians improvise with diatonic tuning. Other equipment included sets of pentatonic boomwhackers (used just by the Synchrony boys, who were particularly active and inventive), a mallet for use by an ‘auctioneer’ in one game, and the texts of various songs and poems written on flipchart paper.

### Procedure

#### Music and Language Sessions

Six sessions of 50–55 min were held weekly, with either five or six children in each group (see Table 1 above). Content was as described in section “Activities” below. Each session’s particular goals and methods were planned ahead, with a specific duration allotted to each activity. As it was important to respond to individual Ps’ interests and to the mood of the moment, plans quite often changed in minor ways, and sometimes a planned activity was omitted. If deviations from the plan meant that a goal had not been achieved, the activity was included in the plan for the next session.

#### Psychometric Testing

Each child was tested individually in a quiet room at their school within 3 days before the first music and language session, and 3–11 days after the last session. Tests were administered by the first author, who holds a degree in psychology and is trained and experienced in psychological testing. After establishing rapport, testing took place in the order TEQ, RMIE, CFSEI-3. Several children struggled with reading some questions, so, for uniformity, each was read aloud unless the child found this annoying. If the child said they did not understand a word or item, it was explained. A neutral presentation and rate of speech were used at all times, each sentence or phrase receiving the same intonation across instances. This was possible because the first author is also a phonetician. Each child sat directly in front of the test paper, with the tester to their left, and wrote their responses independently, without intervention from the tester.

Testing sessions took between 25 and 45 min, with the vast majority lasting about 30 min. Rests were allowed during and between tests, during which the child chatted informally with the tester.

Testing after the last session followed the same procedure. For School1, testing took place 10–11 days after the course (immediately following the week-long half-term holiday), and for School2 it was 3–5 days afterward except for one child. This Turn-taking boy was tested at home 7 days after the sessions finished, because he had moved to live in another town.

Results were scored by the first author after all second tests were concluded. All were triple-checked.

### Activities

#### Methods Common to Both Turn-Taking and Synchrony Groups

For both Turn-taking and Synchrony groups, activities comprised a mix of instrumental and vocal music-making, with or without body percussion, and language games including conversations and related activities, poetry and rapping. Supplementary Table S1 describes activities used in sessions 5 and 6. All session plans are available on request.

A typical session would start with djembe drumming, move on to balaphons with a particular musical or linguistic goal, then alternate between language and musical activities, or activities that involve both. Sessions always finished with free improvisation in which each participant chose which instrument(s) to use. By Session 6, many participants were playing on multiple instruments with impressive invention. Many activities took place with the whole group participating, others with the group divided into (usually two) sections, or in pairs who worked without reference to the other pairs.

The sequence of activities within and between sessions was carefully structured and controlled. Within a session, activities were sequenced to provide variety between music-making and language. Between sessions, the more complex skills needed for later work built on those already learned. Each activity’s basic procedures were first taught and practiced within a well-defined structure until participants were confident, then variation and free work were introduced. When appropriate, underlying principles were briefly discussed, especially in later sessions. Both authors took part, dividing leadership of activities according to their primary skill, music therapy (CF) or speech and language (SH).

Musical activities in early sessions encouraged sensing a regular pulse, counting rests between beat sequences of various lengths, listening to and following each other’s dynamics, following a leader, remembering and reproducing simple rhythmic and/or melodic motifs, and so on. Later sessions introduced more complex rhythms and melodies, and more opportunity for free improvisation, including improvisational singing using vocables, and ensemble work based on 12-bar blues. Thus, at first motifs were provided and there was much imitation, whereas by the end there was a fair amount of improvisation within prescribed conceptual limits.

Language games focused on rhythmic properties of language and conversation. The same principles of progression were used as for the musical activities: language work was initially easy with little or no need to think about content, which was either provided (e.g., receiving and then passing an imaginary ball on beat while saying ‘whish’ on receiving it and ‘whoosh’ on passing it) or fairly automated (e.g., conversations in numbers) as described in section “Differences in methods for Turn-taking and Synchrony groups.” Later, content became more important, but was still kept simple and predictable so that fluent timing could be achieved.

Many activities involved both music and language, to differing degrees. Most obviously, songs, raps, and chants contain both musical and linguistic elements. More inventively, as described below, in several activities normally classed as linguistic, such as reciting a poem, the focus was on maintaining rhythm, ‘melody’ and body movement. Conversely, several musical activities explicitly imitated rhythms and melody of specific utterances, and (for the Turn-taking group) the type of prediction and turn-taking typical of natural conversation.

All songs were familiar at that time (2016): *Eye of the Tiger* (Survivor) and *Rather Be* (Clean Bandit). Raps were initially made up by creating rhythmically interesting motifs from names of football teams, cars, singers, pop groups, etc. each participant choosing and chanting a different name (and hence rhythm) repeatedly. The chants strongly emphasized the words’ particular rhythm, e.g., *Mánchester Uníted*, or *Mán Mán Mánchester, Mánchester Uníted.* The group chanted simultaneously or as a fugue etc, with the Music Therapist controlling individual entries and exits to create an exciting sound. Later sessions used well-known songs/raps, usually *Prince of Bel Air*, and/or limericks, participants following the words on the flip chart as needed. Participants could choose to accompany rapping with clapping and/or percussion instruments *ad lib*., and the Music Therapist accompanied on keyboard.

As the sessions progressed, simple activities (like whish-whoosh, conversations in numbers, musical imitation and counting musical pulses) were dropped and more complex ones introduced. The transition between simple and complex began in Sessions 3 and 4; it was of course gradual and came earlier for some types of activity than others, and for groups whose initial concentration, understanding and behavior allowed faster progress. Once a song or rap was mastered, the words were accompanied by actions and body percussion modeled on Pentatonix’ *White Winter Hymnal^4^*, as well as by the Music Therapist on keyboard.

#### Differences in Methods for Turn-Taking and Synchrony Groups

Activities were designed so that, as far as possible, each turn-taking activity had a synchrony equivalent matched in structure, cognitive and physical demands (Supplementary Table S1). The time spent on musical and language activities was similar between groups. Since the Synchrony group did no natural conversation, they spent longer rapping and exploring natural and exaggerated language rhythms via poetry, whereas the Turn-taking group had more variety in language games because much of their work focused on attributes of conversation. Beyond these differences, there were differences between Treatment groups in how an activity was carried out. The same broad principles differentiating the groups applied to both music and language.

##### Outline of Differences

**Synchrony groups** emphasized keeping a steady pulse, starting and stopping together, following dynamics, and (in later sessions) leading vs. following. These methods amounted to working in fairly standard ways for British music ensemble work, though with more emphasis on improvisation than might occur in most non-therapy situations.

**Turn-taking groups** did all the above activities, and in addition ‘passed’ their turn to others while performing the same or similar activities, and interjected ‘backchannels’ (*uh-huh*, *right on* etc., often with accompanying gestures, and musical equivalents) in ways modeled on natural conversation. Initially, turns were passed in predictable sequence around the group but in later sessions, the current performer chose who to pass to at random, looking and nodding subtly at the next recipient just as they gave up the turn. Thus everyone not performing the main turn had to predict when the current performer would choose to pass the turn on, and be ready to respond with appropriate timing.

##### Detailed Differences

**Synchrony groups** emphasized starting and stopping together, and keeping together with a steady pulse while playing or talking. Participants practiced looking at and listening to a leader, and in later sessions they were taught how to lead such that everyone else joined in or stopped simultaneously, and to adjust their loudness so that an improvising soloist could be heard above the background ensemble (Supplementary Table S1, cells 1S, 6S). Other types of sequential interaction were avoided, but (long) solos were encouraged to equate with the solo time inherent in turn-taking (Supplementary Table S1 cells 2S, 3S, 4S). Language work focused on melodic and rhythmic properties of spontaneous and poetic language, including solos and imitating particular utterances on balaphons (Supplementary Table S1, cell 6S), but avoided anything resembling language interaction between players.

**Turn-taking groups**, in contrast, did a lot of ‘passing’ while performing the same or similar activities: smooth, well-timed transitions between performers were encouraged. Initially, turns were passed in predictable sequence around the group (Supplementary Table S1, cells 3Ti and ii, 4T, 5Tiv, 7Tii), but in later sessions, the current performer chose who to pass to at random, looking and nodding subtly at the next recipient just as they gave up the turn (Supplementary Table S1, cells 1Tii, 3Tiii). Thus everyone not performing the main turn had to predict when the current performer would choose to pass the turn on, so that the pulse and/or talking pace was maintained.

Initially, most turn-taking games involved short turns to minimize cognitive load. For example, in Whish-Whoosh, outlined in section “Methods common to both Turn-taking and Synchrony groups,” the two words were at first prescribed, and later freely chosen within a guiding rule, e.g., they should rhyme, or begin with the same sound. Conversation in numbers, which involves expressing emotion and empathy (or not) without having to think about content or ‘being right,’ allowed more individual freedom because it was easily understood, typically competently executed, and widely enjoyed. So the instruction might be to have an argument and resolve it. The ‘words’ are number sequences (of a length suitable for expressing a particular viewpoint) beginning at 1, so the current talker just starts with the next number in the sequence, and may produce a single number, quite a long string, or something in between; most interlocutors produced a wide range of lengths during the course of a ‘conversation.’

Several other turn-taking games involved one or more interlocutor(s) aligning short responses in time with another person’s longer ones. Examples are: shouting *uh-huh* or *right* and gesturing with the fist in between phrases someone else was singing, bidding at the right time in response to an auctioneer’s patter, and getting the timing of pauses and *uh-huh* right in receiving directions on how to get from one place to another in the school (Supplementary Table S1, cells 2T, 5T, 6T). This task also requires the person giving the instruction to pause after each phrase just long enough to allow the listener to absorb the information, while the person who asked for directions needs to time an *uh-huh* and a nod just right, to allow the next instruction to follow briskly.

Turn-taking in music followed the same principles, and we developed musical analogs of some of the language games. For example, we imitated the ‘directions’ game with balaphons: a soloist improvised continuously while one or more people provided one- or two-note interjections at phrase boundaries when they judged it to be appropriate (Supplementary Table S1, cell 6T). This task was valuable for all players: the soloist had to produce clear but not exaggerated or unmusical phrase boundaries, which was sometimes hard, and others had to predict when the phrase would end so that they could interject. Additionally, with djembe drumming especially, there was an emphasis on traditional call-and-response forms (Supplementary Table S1, cell 1T). Similarly, words and accompanying actions were passed around the group, initially in sequence around the circle of participants, and sometimes in later sessions by the current performer looking and nodding at the chosen recipient just as they gave up the turn (Supplementary Table S1, cell 1T, 7T).

#### Tailoring Methods to Groups and Individuals

Time spent on specific activities varied somewhat between groups for several reasons. The dynamic within each group was unique, and individuals within groups also differed from one another. Girls’ groups were generally quieter and more compliant than boys’, but each group had individual exceptions. Additionally, groups differed slightly in their competence at some activities, enjoyed them to differing degrees, or preferred different types of content for the same basic activity (especially poems and songs). Over all sessions, however, every effort was made to give all individuals the same general experiences and time spent on them.

In sum, members of both groups acquired similar motoric skills, and learned to listen, be creative and to perform confidently in front of their peers. The Synchrony group also learned to maintain a good rhythm, to lead and to conform (follow). Their leading required more self-reliance than sensitivity to peers. The Turn-taking group acquired the more subtle rhythmic and gestural skills associated with turn-taking in successful conversations and ensemble improvisation in music. These skills required attention to and accurate prediction of another person’s behavior, together with tight timing of a coordinated but distinct response.

## Results and Preliminary Discussion

### Attendance

Table 2 shows the average number of sessions attended (range in parentheses), for each subgroup. Twelve of the 22 children achieved 100% attendance. Most sessions missed were unavoidable: hospital appointments, illness, pre-arranged school trips, or examinations. Attendance is not factored into the main analyses since patterns were fairly well balanced across both Treatment Group and Sex.

**TABLE 2:** Average (range) of participants’ attendance at a maximum of six sessions.

	Treatment Group				
	Turn-taking		Synchrony		Whole group
School	School1	School2	School1	School2	
Female	5.4 (3–6)	–	–	4.8 (3–6)	5.09
Male	–	5.0 (4–6)	5.4 (4–6)	–	5.18
Whole group	5.18		5.09		

### Overview of Statistical Analyses

#### Analyses

The main issue of interest is whether the Turn-taking group changed positively more than the Synchrony group. This is reflected in the difference between psychometric test scores obtained before and after the sessions, hereafter called Change scores. Also of interest is whether girls responded differently to the treatments than boys. Interpretation of Change scores requires us to also ask whether there were differences between the groups before the sessions started, and whether observed changes were significantly different from zero.

Shapiro–Wilk normality tests were conducted using R (shapiro.test) on Before, After, and Change scores for every test. TEQ and RMIE scores were all normally distributed (*p* > 0.05), as were most Self-Esteem scores. Five Self-Esteem subscores were not normally distributed (*p* < 0.03). Of these, Defensiveness After the sessions was skewed toward 0 (*W* = 0.873, *p* = 0.009). This skew indicates that participants answered honestly. We assume their honesty generalizes across all self-reports.

The non-normality of the other four self-esteem distributions encouraged supplementary use of non-parametric statistics for these cases: Academic and Social Before subscores; Parental/home and Social After subscores. Finally, the Change score for General Self-Esteem just missed the standard value for acceptance (*W* = 0.912, *p* = 0.053). Since in tests for normality it is more conservative to make no correction for multiple tests, this result can also be taken as encouraging the use of non-parametric statistics.

For completeness, therefore, and as a reliability check, all results underwent the following analyses: Before, After and Change scores for Treatment, Sex, and their interaction, using 2 (Treatment Groups) × 2 (Sex) between-groups univariate ANOVA conducted in SPSS (v22), with additional one-tailed Mann–Whitney *U* tests (Wilcoxon tests for independent samples) in R (v 3.2.2, wilcox.test). One-sample *t*-tests were used to test whether mean change scores differed significantly from zero, using two-tailed p levels, although since positive change was predicted, one-tailed tests could be justified.

The standard significance level of *p* ≤ 0.05 was adopted, and (in recognition of the relatively small numbers of participants and the exploratory purpose of the study) results with *p*-values of about 0.07 are flagged as marginal and discussed when the pattern of data suggests they merit attention. Bonferroni-corrected *post hoc* tests were used to explore interactions when patterns of change indicated they were needed (Howell, 2010, p. 366).

No corrections were made for multiple comparisons across the three tests, since they were considered independent, nor within the CFSEI-3. This is acceptable for TEQ and RMIE, each of which produces only one score. It is incautious for CFSEI-3, which produces six subscores analyzable here (People > 13 years have a 7th subscore, ‘Personal’; these results were not analyzed.) However, observed p levels were such that correction would not change conclusions.

#### Data Quality

All tests were completed satisfactorily with one exception: the RMIE After score for one Turn-taking girl. Prolonged noise in the testing area by a visiting school seriously distracted her, and it proved impossible to test her later. The disruption being unexpected, the data point was deemed missing ‘completely at random’ (Cohen et al., 2003, p. 433). To preserve the same power across all tests, the value was estimated using two methods in SPSS, MVA regression, and imputation modeled on the Turn-taking group, Predictor = Before scores, Predicted = After score. Both methods converged on a Change score of +2.

### Empathy

#### Toronto Empathy Questionnaire: Self-Report

**FIGURE 1:**
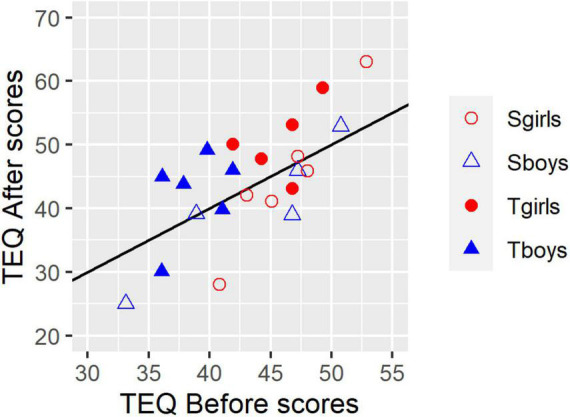
Toronto Empathy Questionnaire Before and After scores for each of 22 participants. Symbols indicate subgroups. Filled symbols: Turn-taking. Open symbols: Synchrony. Red circles: girls. Blue triangles: boys. Datapoints with identical values are slightly offset on the *x*-axis so they can be seen. The solid black diagonal line shows *x* = y, i.e., a symbol appearing on this line indicates no change between Before and After Scores. Higher After than Before scores lie above the line, and lower ones lie below the line.

The 16-item TEQ has minimum and maximum scores of 0 and 64 respectively, with extreme scores unlikely. In the populations of 18-year-old Canadian undergraduates on which the test was normed, mean scores were around 45 (SDs around 7.5). Figure 1, a scattergram that represents each participant in terms of their Before (*x* axis) and After (*y* axis) scores, shows that the present results compared satisfactorily with reported norms.

**FIGURE 2:**
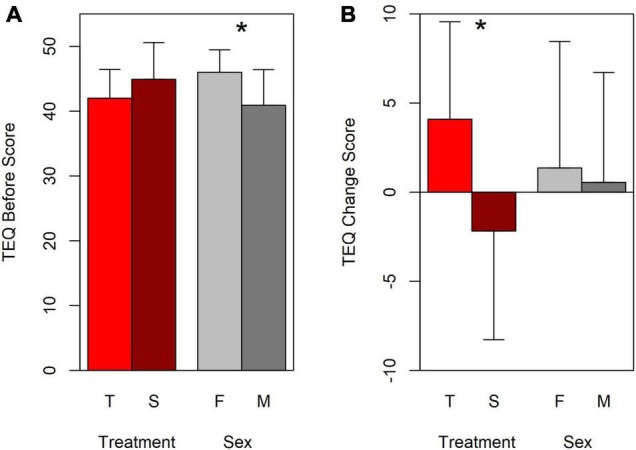
Mean scores and standard deviations for TEQ Before **(A)** and Change **(B)**. Red shaded bars: mean scores for Treatment group, T, Turn-taking; S, Synchrony. Gray shaded bars: mean scores for Sex, F, Female; M, Male. Error bars = standard deviation. Positive Change scores represent improved performance (After > Before). * Between bars: *p* < 0.025 in ANOVA.

Figure 2A shows the means and standard deviations for empathy Before the intervention, for Treatment Group and Sex separately. There was no difference between the treatment groups at outset [mean Before scores: Turn-taking 42 (SD 4.43), Synchrony 45 (SD 5.67), *F*(1,18) = 1.643, *p* = 0.216, $\eta_{\text{p}}^{2}$ = 0.084; *W* = 96.0, *p* = 0.991], but as expected girls scored slightly higher than boys [mean scores: Girls 46 (SD 3.46), Boys 41 (SD 5.49), significant in the ANOVA, *F*(1,18) = 6.384, *p* = 0.021, $\eta_{\text{p}}^{2}$ = 0.262] but not the Mann–Whitney *U* test (*W* = 96.0, *p* = 0.991). The Treatment × Sex interaction was not significant (*p* = 0.3). The significance of the Sex difference needs interpreting cautiously, possibly in favor of non-significance, given the discrepancy between the two tests. The appropriateness of the ANOVA is arguable: the Shapiro–Wilk test of normality on the whole data set is not significant (*W* = 0.976, *p* = 0.848), but the variances are not homogeneous (Levene’s test, *p* = 0.028), although the difference is small and Hartley’s Fmax = 7.95 suggests that they could be treated as homogeneous. The important point, however, is that there was no initial self-reported empathy difference between Treatment groups.

In contrast, **TEQ change scores**, shown in Figure 2B as deviations from 0 (no change) were significantly greater for the Turn-taking group compared with the Synchrony group on both ANOVA and Mann–Whitney tests: mean change = 4.09 (SD 5.47) and –2.18 (SD 6.10) for Turn-taking and Synchrony groups respectively [*F*(1,18) 6.095, *p* = 0.024, $\eta_{\text{p}}^{2}$ = 0.253; *W* = 28.0, *p* = 0.018]. There was no interaction between Treatment group and Sex (*p* > 0.9). The Turn-taking group’s mean change score was also significantly greater than 0 [two-tailed one-sample *t*-test *t*(10) = 2.48, *p* = 0.032], whereas the Synchrony group’s was not [*t*(10) = –1.19, *p* > 0.2]. There was no difference between the sexes in amount of change (*p* > 0.5, gray bars). The pattern of Figure 2B summarizes what is also evident in Figure 1: most Turn-taking points lie above the *x* = y line (represented as a positive change in Figure 2B), whereas few Synchrony points do (indicating a zero or negative change).

In sum, the Turn-taking activities reliably enhanced mean self-reported empathy, whereas the Synchrony group’s activities had no consistent effect.

#### Reading the Mind in the Eyes Test (Child): Behavioral

**FIGURE 3:**
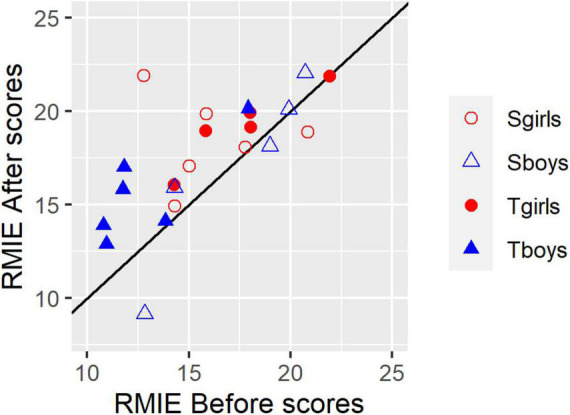
Reading the Mind in the Eyes Test Before and After scores for each of 22 participants. Symbols indicate subgroups. Filled symbols: Turn-taking. Open symbols: Synchrony. Red circles: girls. Blue triangles: boys. Datapoints with identical values are slightly offset on the *x*-axis so they can be seen. The solid black diagonal line shows *x* = *y*, i.e., a symbol appearing on this line indicates no change between Before and After Scores. Higher After than Before scores lie above the line, and lower ones lie below the line.

The 28-item behavioral test of Reading the Mind in the Eyes (Child) reports mean scores of about 20 (SD 2.4) for 19 typically developing 10–12 year old children: 20.2 (SD 2.4) for 9 males, 21.0 (SD 2.4) for 10 females (Baron-Cohen et al., 2001b). Scores become progressively lower for younger children. Our participants’ scores could be expected to be 20 or more. However, mean score for the entire group was relatively low: before the sessions, 16 (SD 3.41), girls 17 (SD 2.89), boys 15 (SD 3.77); after the sessions 18 (SD 3.26), girls 19 (SD 2.23), boys 16 (SD 3.72). Figure 3 confirms that few participants scored more than 18, especially Before the sessions.

**FIGURE 4:**
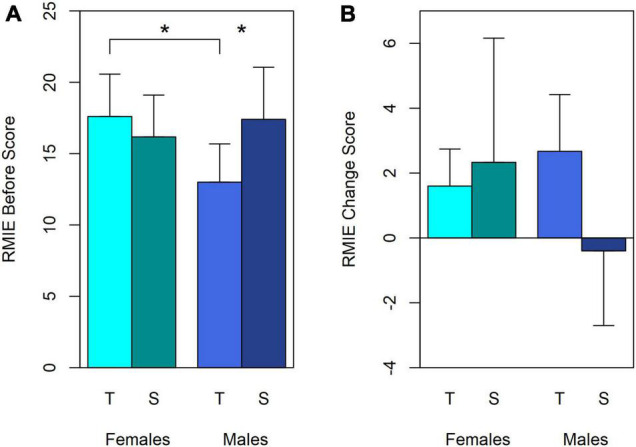
Mean scores and standard deviations for the interaction between Treatment groups and Sex in RMIE Before **(A)** and Change scores **(B)**. Cyan bars: mean scores for Females. Blue bars: mean scores for Males. T, Turn-taking; S, Synchrony. Error bars are the standard deviation. Positive Change scores represent improved performance (After > Before). *p < 0.03 in ANOVA.

ANOVAs of Before scores showed a significant interaction between Treatment group and Sex [*F*(1,18) 4.99, *p* = 0.038, $\eta_{\text{p}}^{2}$ = 0.217]. There were no other significant differences within Before, After, or Change ANOVAs. Figures 4A,B show the mean Before and Change data respectively, but arranged differently from the equivalent TEQ data in Figure 2, to clarify the Treatment Group and Sex interaction.

The interaction between Treatment Group and Sex in the Before scores (Figure 4A) was significant because Turn-taking boys began less skilled than the other three subgroups: their Before scores were only 13 (SD 2.68), whereas the other three subgroups scored 16-18 (SDs 2.93–3.65). *Post hoc* Bonferroni-corrected comparisons confirmed that these differences are significant: Turn-taking boys scored lower than Turn-taking girls [*F*(1,18) = 6.21, *p* = 0.023, $\eta_{\text{p}}^{2}$ = 0.257] and Synchrony boys [*F*(1,18) = 5.683, *p* = 0.028, $\eta_{\text{p}}^{2}$ = 0.240]. Indeed, they were within the range for 20 typical 6–8 year olds, as well as for 15 boys (8–14 years) diagnosed with Asperger syndrome for whom Baron-Cohen et al. (2001b) report a mean of 12.6 (SD 3.3). Recall that the present study excluded children with diagnoses or clear traits of autism.

Figure 4B shows the RMIE Change scores. Scores improved for all subgroups except Synchrony boys. The change was significantly greater than zero for the group as a whole [mean difference 1.64 (SD 2.63), one-sample *t*(21) = 2.92, *p* = 0.008, two-tailed], and for the Turn-taking group alone [mean difference 2.18 (SD 1.54), *t*(10) = 4.71, *p* = 0.001]. The Synchrony group alone did not improve significantly [mean difference 1.09 (SD 3.39), *t*(10) = 1.07, *p* = 0.31].

Turn-taking boys improved disproportionately. With a mean After score of 16 (SD 2.58), they reached almost the same skill level as the other three groups [means 17–19 (SDs 2.17–5.00)]. Consequently, the Treatment group × Sex interaction, significant in the Before analysis, was reduced to non-significance in ANOVAs for both Change [*F*(1,18) = 3.08, *p* = 0.096, $\eta_{\text{p}}^{2}$ = 0.146] and After analyses [*F*(1,18) = 0.60, *p* = 0.464, $\eta_{\text{p}}^{2}$ = 0.030], and no main effects were significant (Change *p* ≥ 0.3; After Treatment group *p* > 0.8, Sex *p* > 0.08).

The individual RMIE data suggests participants with low initial scores benefited most from the program: Figure 3 shows a trend across all groups for large changes amongst participants with low initial scores, and smaller ones for participants with high initial scores: with only one exception, relatively low initial scores (16 or less) fall on or above the *x* = y line, while high ones (18 or more), tend to be close to the line and more evenly distributed around it. Since the maximum score is 28, high scorers seem unlikely to be subject to ceiling effects. Turn-taking boys (filled blue triangles) and Synchrony girls (open red circles) comprise most of the low-scorers.

The one exception mentioned is a Synchrony boy who scored 13 Before but only 9 After the sessions. His score largely accounts for the slight negative change of the Synchrony boys group (Figure 4B). The overall trend also confirms why there were no significant differences between groups in the Change scores, and particularly that the Turn-taking boys, all but one of whom were amongst the lowest scorers Before the sessions, improved so much in comparison with the others that the interaction observed for the Before scores was no longer significant in the After scores. Likewise, while the mean change score for Synchrony girls was relatively large, so was this group’s variance (Figure 4B): Synchrony girls who scored relatively low Before the sessions (Figure 3) improved appreciably, whereas the two who scored highest did not.

In summary, RMIE scores improved overall between starting and ending the sessions, with the Turn-taking group gaining most. Children who initially scored low tended to improve more than children who initially scored high.

### Self-Esteem (Version 3 of the Culture-Free Self-Esteem Inventory)

#### Change in Self-Esteem

**FIGURE 5:**
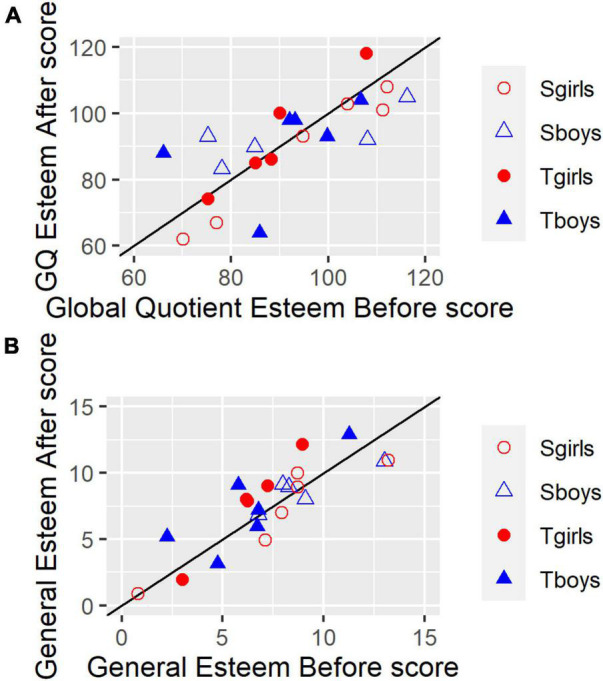
CFSEI-3 Before and After scores for each of 22 participants. **(A)** Global Quotient. **(B)** General Self-Esteem subscale. Symbols indicate subgroups. Filled symbols: Turn-taking. Open symbols: Synchrony. Red circles: girls. Blue triangles: boys. Datapoints with identical values are slightly offset on the *x*-axis so they can be seen. The solid black diagonal line shows *x* = *y*, i.e., a symbol appearing on this line indicates no change between Before and After Scores. Higher After than Before scores lie above the line, and lower ones lie below the line.

The CSFEI-3 self-esteem test produces an overall Global Quotient (GQ) and separate scores for several subscales (four for 12-year-olds, five for older children). Scores are standardized. The GQ has a mean of 100, standard deviation of 15, and standard error of 5 (12-year-olds) and 4 (other ages). Each subscale has a mean of 10, standard deviation of 3, and standard error of 1 (2 for 12-year-olds’ General subscale). Battle (2002, p. 15) describes the Global Quotient as the most useful of the CSFEI-3 scales, and suggests subscale scores should be used only when speculating about why the Global Quotient is low. Consequently, Figure 5A shows individual data for the Global Quotient, while Figure 5B shows data for the General self-esteem subscale. These two scales achieved the most statistically reliable Change differences between Treatment groups. [All the scales are highly reliable. Test–retest reliability for the GQ is 0.86 and 0.98 for the Intermediate and Adolescent forms respectively, and over 0.90 for most subscales (Battle, 2002, Tables 5.5, 5.6).]

Figure 5 shows considerable variation between participants in the present study, but scores tended to be lower than average, resulting in a mean GQ in the low 90s for all subgroups, and subscale means ranging between 6 and 8.5, except for the Personal/Home subtest, whose means were around 11 (toward the high end of average). There were few significant differences between groups, so for simplicity only some of the data is discussed in detail.

Standardized scores outside the mean ± 10 are considered as “warranting diagnostic attention.” That is, while low scores are obviously problematic, very high scores are not necessarily considered better than average scores: they could indicate one of several disadvantageous situations, like misplaced confidence or opting to give a good impression. Given that these participants were chosen as socially vulnerable and most were anxious, the sign of GQ Change scores was reversed when the Before score was 115 or higher and the After score was smaller—i.e., a fall from a very high score was treated as positive for analyses involving Change. The equivalent cutoff for subscales was 13.

**FIGURE 6:**
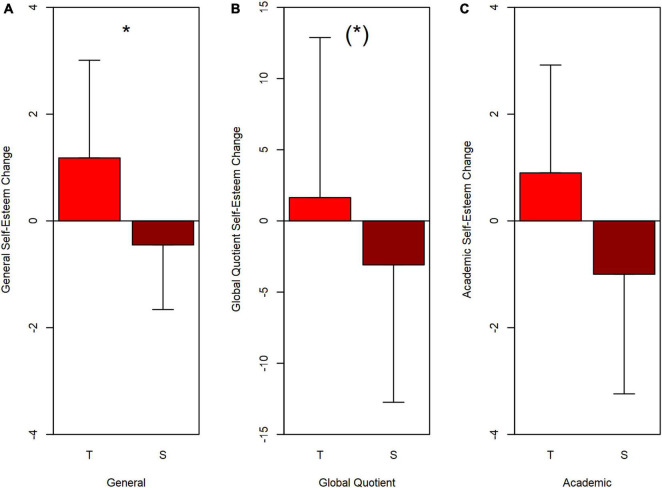
Mean Change scores and standard deviations for three scales of the CFSEI-3, arranged in order of statistical significance between Treatment groups. **(A)** General subscale; **(B)** Global Quotient; **(C)** Academic subscale. T, Turn-taking group; S, Synchrony group. Positive Change scores indicate improved performance. *p = 0.027 in ANOVA. (*) *p* = 0.073 in Wilcoxon test.

There were no statistically significant differences in self-esteem between Treatment groups or Sex before sessions began. Change scores likewise showed few significant main effects and no significant interactions. Figure 6 shows the three largest Change main effects.

Amongst the Change scores, only General self-esteem (Figure 6A), which measures a person’s overall perceptions of self-worth (Battle, 2002, p. 15), showed a significant difference between treatment groups over the testing period, with greater improvement for the Turn-taking group than the Synchrony group. For Turn-taking and Synchrony respectively, the mean General Change scores were 1.18 (SD 1.83) and –0.45 (SD 1.21) [*F*(1,18) = 5.79, *p* = 0.027, $\eta_{\text{p}}^{2}$ = 0.243, Wilcoxon (Mann–Whitney) *W* = 28.0, *p* = 0.017]. As detailed below, the Turn-taking group’s positive Change was significantly different from zero, whereas the Synchrony group’s negative change was not.

Figure 6 also shows changes for the Global Quotient (B), and Academic self-esteem (C). Though the predicted trends for greater positive change for Turn-takers were evident, only the GQ change even approached statistical significance. GQ: Turn-taking mean 1.64 (SD 11.24), Synchrony mean –3.09 (SD 9.65), (Mann–Whitney *W* = 38.0, *p* = 0.073). Academic: Turn-taking mean 0.09 (SD 2.02), Synchrony mean –1.0 (SD 2.24), *W* = 40.0, *p* = 0.091. (ANOVAs were not run for these two scales due to non-homogeneous variances.)

Changes from zero were also assessed, using both two-tailed one-sample *t*-tests and one-sample Wilcoxon tests. Only the Turn-taking group’s General subtest was significantly different from zero [mean 1.18, *t*(10) = 2.14, *p* = 0.058; *V* = 47.00, *p* = 0.048], which is of course in the predicted positive direction. All the rest fall far short of significance (two-tailed *p* > 0.7 for Turn-taking, and *p* > 0.17 for Synchrony).

In summary, Turn-taking significantly enhanced General self-esteem, had unreliable (weak) positive effects on GQ self-esteem, and no effect on the other subtests. Synchrony experiences had no significant effect on self-esteem.

#### Defensiveness

Defensiveness assesses honesty of answering, so (unlike the other tests) a low Before score, and/or a negative change over time, are desirable. Defensiveness scores were typically low on first testing: means of 2.64 (SD 1.69) and 2.73 (SD 2.01) for Turn-taking and Synchrony respectively, both well below the limit allowed for answers to the rest of the questionnaire to be deemed honest (6 for ≤12 years, 4 for ≥13 years).

**FIGURE 7:**
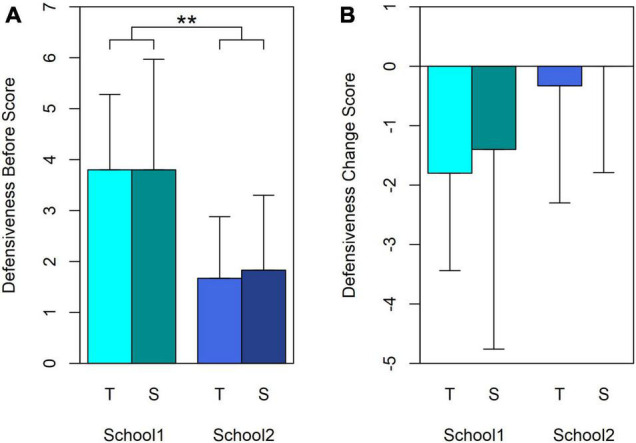
Mean scores and standard deviations for the interaction between Treatment groups and Sex in the Defensiveness scale of the CFSEI-3. **(A)** Before scores. **(B)** Change scores. The data are arranged to highlight differences between Treatment groups and schools, rather than Sex. Cyan bars: mean scores for School1. Blue bars: mean scores for School2. T, Turn-taking; S, Synchrony. Asterisks between bars for the two schools mark the significant interaction in the Treatment group × Sex ANOVA (*p* = 0.008). Unlike other tests in this study, a low score is preferable, and hence a larger negative Change shows improvement (less Defensiveness).

The Treatment group × Sex ANOVA of the Before scores showed no significant main effects but a strongly significant interaction [*F*(1,18) = 9.015, *p* = 0.008, $\eta_{\text{p}}^{2}$ = 0.334]. This was caused by Turn-taking girls and Synchrony boys being more defensive than the other two groups. Table 1 confirms that school rather than Treatment or Sex accounts for this interaction. Figure 7A shows the Before data, arranged by Treatment group within School to highlight the School difference. School1 children were almost twice as defensive at outset as School2 children [*t*(20) = 0.007, two-tailed Mann–Whitney *U*, *p* = 0.009].

Changes in Defensiveness were largely as expected: zero (2) or negative (15) for 17 of the 22 participants, and positive for the remaining 5. Figure 7B shows the Change scores for Schools and Treatment groups. School1 participants changed in the (desirable) negative direction to a greater extent than School2, but the difference is not statistically significant [*t*(16.001) = –1.514, *p* = 0.15, two-tailed, equal variances not assumed; Mann–Whitney *U*, *p* = 0.107], perhaps due to a floor effect for School2, whose Before scores were already low. The Change scores reflect the fact that, although the After scores maintained the same pattern as the Before scores, the differences between subgroups were smaller in the After scores, resulting in no significant main effects or interactions in separate Treatment groups × Sex, and Treatment groups × Schools ANOVAs.

In summary, School1 children were more defensive than School2 children before the study, but by the end they reduced that difference sufficiently to produce statistically non-significant differences.

## Discussion

### Overview

This study shows that practice in turn-taking while doing group activities in music and language can improve psychometric test scores on empathy, and to some extent self-esteem, amongst 12–14 years old who struggle in social situations (but do not have notable autistic traits).

The greatest measured benefit was for empathy. Empathy increased in self-report (TEQ) and in accuracy of reading emotions from a person’s eyes (RMIE). For both tests, the absolute degree of change is significantly greater than zero for Turn-taking but not Synchrony groups. Synchrony groups as a whole did not change significantly, despite their activities being similar except for the Turn-taking element. However, for the behavioral RMIE, low scorers benefited more from the program than high scorers, regardless of Treatment group, though practice in turn-taking benefited most participants too.

Turn-taking enhanced self-reported self-esteem (CFSEI) less reliably than it enhanced empathy, but nonetheless encouragingly. The only statistically significant change in CFSEI scores was in the General subtest. For this, the Turn-taking group improved relative to their scores on first testing and relative to the Synchrony group. For the Global Quotient, the Turn-taking group improved marginally better than the Synchrony group (*p* = 0.073). Non-significant trends in the other tests patterned consistently in favor of greater benefit from activities that include turn-taking. When the training has been relatively short, it is perhaps a lot to expect large changes on a test with high test–retest reliability. Nonetheless, it is interesting and reassuring that the most reliable effect was in General self-esteem, rather than, e.g., Social or Academic, which would take time for the testee to accumulate evidence for.

Although there were differences in how girls and boys behaved in their groups, boys generally (but not uniformly) being livelier, the only sex difference that affected the Treatment group results was for RMIE. Low-scoring boys improved disproportionately, which, given the study design, affected the overall group statistics. Baron-Cohen et al. (2001a) report a mild correlation between boys and low scorers, as found in the present study. We also found one for girls. We tentatively conclude that, used responsibly, the greater interpersonal understanding gained from games involving musical and linguistic turn-taking during meaningful interaction will in time increase empathy and the possibility for more prosocial behavior in teenagers of both sexes.

### Contribution of the Current Study

The hypothesis was developed by drawing novel connections between the literatures of music, rhythmic entrainment, and the phonetics of conversation. The intervention program involved four treatment groups and lasted 6 weeks. Group size was only six, since participants needed individual attention to maximize both learning and enjoyment. Consequently, this first test was necessarily small-scale. The results encourage further investigation with more participants and a wider range of vulnerable populations, and perhaps more sessions. For example, although Fancourt et al. (2016a) showed that self-selected older adults, who underwent 6 weekly 90-min sessions of group drumming, benefited on measures of physiology, mental health and well-being, compared with matched controls who participated in social but not musical activities, just 5–6 one-hour sessions is probably the minimum to effect measurable improvement on psychometric tests, which are designed to have high test–retest reliability.

Details may not replicate due to study size, but since the results are consistent overall, the general patterns seem likely to. For example, while improvement in self-report measures need not necessarily result in behavioral changes, and can presumably be fudged (notwithstanding high test–retest reliability), improvements in a measure like the RMIE seem likely to be less subject to testee bias, and more likely to generalize to measurable benefits in social situations. Thus these results seem worth following up with larger groups and more sessions.

Put more generally, turn-taking modeled on natural conversation and applied in music and language games takes time to learn and is a more subtle investigative manipulation than, for example, performing music or not, or acting with shared rhythm or not. So we would expect our turn-taking manipulation to take time to show measurable benefits in empathy and self-esteem. This is especially true given that as far as possible the activities were identical except for the turn-taking element. In sum, although greater change would have been more convincing, the measured effects may be about what can be expected with a short program and small sample sizes, especially given the sensitivity of these children to difficulties in their everyday lives (see e.g., section “Why little or no change for the Synchrony group?”). These caveats on the reliability of the results noted, the following sections explore their potential.

### Why Little or No Change for the Synchrony Group?

Although no mean Synchrony group changes were significantly different from zero, that they were consistently negative is puzzling. There is no evidence it is due to skewing of means by outliers. Yet benefits were both predicted from the literature on effects of shared rhythmicity [see section “Introduction” for self-esteem and prosocial behavior, and Hallam and MacDonald (2009) for other benefits], and observed during sessions—all children appeared to enjoy themselves, and gained noticeably in musical skills, creativity, and performance confidence.

Negative scores tended to be more common amongst School2 children: Turn-taking boys and Synchrony girls. Their post-session tests took place in the context of end-of-year examination stress and disappointments connected with sports days. These disruptions were noticeable, and seem the most likely explanation for the results. But negative change in self-esteem could occur if the children felt unusually confident on first testing. This might have happened for a variety of reasons: being chosen for the work, knowing they were contributing to research, anticipation of the coming activities and/or the payment, wanting to portray themselves as competent to an unfamiliar person. Teachers commented on these reactions. Such excitement would be less compelling by the end. But if this were the case, then one could expect CFSEI Defensiveness to be higher Before the sessions than After them. But School2 children, whose CFSEI scores changed most in the negative direction, were undefensive at the outset and the end, with no overall change in defensiveness.

The tentative conclusion is that the Synchrony group’s consistent trend for negative change is probably not relevant, especially since none are significantly different from zero. If it does reflect a true difference, it is probably due to end-of-year stress, which these vulnerable children experienced as especially disruptive.

### What Is Especially Beneficial About Explicit Practice in Turn-Taking?

The hypothesis was that experience in turn-taking benefits empathy and self-esteem more than engaging in activities that are essentially identical except that they do not involve turn-taking. Arrived at by combining evidence from disparate research fields whose common factor was entrained rhythm, factors underlying that hypothesis now merit closer scrutiny. How might turn-taking during rhythmic activities enhance mutual attention more than similar activities carried out in synchrony? Possibilities include how participants looked at others, and the role of prediction. Although this study cannot speak directly to either issue, each is discussed briefly to facilitate future research.

Turn-takers were instructed when and how to look at others, whereas there were no such instructions for Synchrony participants. It could be worth assessing total looking time, but it is the function and predictability of looking which clearly distinguishes the treatment groups. For Synchronizers, looking during an activity might help or be rewarding, but since the focus was on keeping the activity going as a group, everything except the start and perhaps the end of the activity could be done auditorily by attending to the beat. For Turn-takers, the behaviors taught for smooth turn passing (eye contact, head nods and other facial and body language), served a communicative function essential to the smooth accomplishment of the activity. Eye contact and body language were integrated into the joint action such that their function was essential to success. In skilled (later) Turn-taking activities, prediction was deliberately made difficult: soloists could pass their turn to anyone in the group, and could vary the number and length of phrases before passing the turn. Meanwhile, potential recipients tried to predict whether they would be chosen: they really were trying to ‘read’ the soloist’s intentions. In short, Turn-takers had explicit instruction in dynamic, temporal attributes of cooperative interaction, and in later sessions gained much more practice than Synchronizers in predicting the intentions of a soloist, as well as in precisely timing brief supportive interjections in both music and language. Novembre et al. (2019) argue convincingly that the relationship between temporally-coordinated musical behavior and social cognition is bidirectional, and better mediated by individuals with high rather than low empathy. Our methods explicitly taught these techniques, and resulted in gains in empathy.

The distribution of ‘functional’ looks also merits investigation. Turn-takers gained much experience in functional looking toward everyone in the group, whereas Synchronizers probably looked most to just the two adult leaders. Directing functional looks to more people may enable individuals to gain empathetic skills that generalize better to new situations. Experiments show that learning from a variety of instances tends to be harder at first but to generalize better to the natural world. For example, when initially exposed to a variety of positive instances, Japanese adults learn to distinguish /r/ from /l/ more accurately in new words and voices, compared with listeners who hear a small set of instances during initial learning (Lively et al., 1993; Pisoni et al., 1994). Learning with a small set allows faster initial classification, but generalizes poorly to the wider variation of natural speech. Likewise for learning sound contrasts from richly varying natural speech versus the unvarying but impoverished cues of synthetic speech (Duffy and Pisoni, 1992). The same principles account for the strides made in artificial intelligence performance after ‘big data’ became available. Extrapolating then, compared with Synchrony tasks, Turn-taking tasks may provide richer, more meaningful (i.e., functionally relevant) experience in processing and responding to auditory and visual cues that are essential for maintaining tight mutual coordination of independent communicative roles.

If practice in functionally-important turn-taking is the crux of the difference between the two groups, it may be critical that performers act with tight interpersonal coordination, yet somewhat independently, typical of conversational turn-taking. In every activity, turn-taking individuals had distinct roles, performing independent parts, often in sequence rather than simultaneously, to produce a whole in which each part was crucial. Consequently, individual Turn-takers typically played/spoke less and listened more to peers than Synchronizers did. Synchronizers’ sound benefited from better listening and cooperation, but there were fewer distinct individual roles, imitation was often feasible, and while it was important to keep the beat, follow dynamics and attend to beginnings and ends, coordinated sequential timing of distinct behaviors between individuals was never required. A heightened ‘sense of responsibility’ perhaps encouraged Turn-takers to work harder at understanding others, but that alone cannot directly increase empathy. Interpersonal coordination plausibly did, and merits further research.

These arguments support the tentative conclusion that, when joint rhythmic activity includes judging another person’s intentions and coordinating one’s own distinctive actions with that person, empathy and to some extent self-esteem increase more than when such rhythmic coordination mainly involves the mutual accommodation necessary to *maintaining* a beat. This conclusion is congruent with Woolhouse et al.’s (2016) evidence that, in silent disco dancing, entrainment to a shared tempo increases eye-gaze and accuracy of memory about interactants who danced to the same beat, but not about people who danced to a different beat.

### Different Routes to Enhanced Empathy and Self-Esteem?

Section “What is especially beneficial about explicit practice in turn-taking?” suggests that the Turn-taking group’s improvement in empathy and aspects of self-esteem was because interpersonal understanding, and hence empathy, develop directly from reciprocally responsive, entrained focus on another person or people doing related but distinct things from oneself. Presumably enhanced (appropriate) empathy with others brings its own social rewards, which in time may enhance self-esteem.

Yet the literature indicates that joint activities which are mainly synchronous enhance a sense of personal well-being within the individual, and lead to prosocial behavior. The sense of well-being presumably precedes the prosocial behavior initially, the two becoming mutually reinforcing. But prosocial behavior (i.e., ‘helping’) does not inevitably produce desirable outcomes. It has positive social consequences only if it is well judged and hence wanted by its recipients. Prosocial behavior without empathy may be unwanted and cause annoyance. When a person acts ‘prosocially’ but ignores or misunderstands how their behavior affects the recipient, the social consequences are likely to decrease empathy.

Thus joint synchrony without reciprocity or the need to continuously monitor other people’s responses, which characterizes the Synchrony group’s games, may enhance empathy more slowly and indirectly than does turn-taking experience. This explanation is compatible with Schellenberg’s (2004) findings that 6-year-olds gained more in adaptive social skills (assessed by parental report) when assigned to drama than to music classes. (For a review see Schellenberg and Weiss, 2013).

### Novel Contributions and Suggestions for Future Work

Two novel contributions of the present study are, first to combine language, music, and ‘cross-over’ activities such as rap in ways that emphasize their common elements, and second, to control activities so that a specific hypothesis is tested, yet in a setting that in all other respects is natural, thus making the methods suitable for therapeutic interventions. These qualities distinguish the present study from others demonstrating social and personal benefits of joint music-making [see section “Introduction,” also Clift and Hancox (2001), drama and language games (e.g., Jindal-Snape and Vettraino, 2007; Jindal-Snape et al., 2011) and Schellenberg’s (2004) control group] or both [Kirschner and Tomasello (2010) and Ex Cathedra’s excellent Education section^5^ ]. This section describes and explains the distinctive elements.

This mixture of rigorous hypothesis-testing in multiple, structured sessions in which the emphasis is on spontaneous behavior rather than scripted interaction, required many aspects of normal experimental control to be relaxed. For example, it was essential to keep participants engaged during each session and eager to return to future ones. Likewise, everyone must understand the instructions and want to contribute. Consequently, in contrast for example with Kirschner and Tomasello’s (2010) creative but highly controlled single-session experiment, much of what we said was specific to the particular children, and whatever children said or did was responded to. Our instructions were precisely worked out and adhered to, but most language used, and the music played, was responsive to particular children’s contributions, interests, and creativity. To enable naturalistic turn-taking, this element of naturalness seems likely to prove essential.

This was a small-scale ‘proof of concept’ study conducted within a small budget. Replication would be advantageous with blind psychometric assessment, and controls on who delivers the program—ideally, more than two leaders and more groups, to remove effects of leader(s). Leaders probably cannot be kept ignorant of the hypothesis, for in our experience, they must understand what activities are or are not possible within each Treatment group. But distributing leaders across groups should minimize unwanted leader effects. Objective measures of behavioral change would be preferable to self-report.

Ideally, replication should widen the age range through to old age. The literature on benefits of joint music-making finds similar effects across the life span, and specific content is easily adjusted to individual interests and abilities. Pilot work showed our methods appealed to older vulnerable teenagers and typical adults. Indeed, compared with younger teenagers, older teenagers were more sensitive to, and better able to manipulate, musical and especially conversational timing and poetic meter. Children younger than about 12 years may benefit less. Rhythmic stability is commonly established only after puberty, and some conversation work demands a good sense of pragmatics. So despite toddlers and young children engaging in turn-taking, the program might need significant modification to work within the rhythmic and cognitive abilities of children younger than about 12, and even then benefits might be small.

The range of individual vulnerabilities may also be wide: music therapists have suggested the program could help people who have depression, including post-natal, teenage parents at risk of neglecting their infants, perpetrators of domestic abuse, some prisoners and ex-prisoners, anorexics, adopted people (many of whom suffer low self-esteem), and carers of people who have dementia.

While the present study combined music and language work, future research might examine effects of turn-taking practice in the two domains separately. An informal impression was that more advanced language work was sometimes tiring and/or less immediately engaging for all. While one would not want to omit language work (and drama in general) from this type of program, and it was usually easy to increase interest and engagement, language work is not suitable for everyone, so it would be worth assessing the merit of turn-taking in structured, interactive musical activities alone, and of linguistic turn-taking games alone.

## Conclusion

This study’s results suggest that interaction that requires looking, listening, predicting and turn-taking seems to particularly encourage the development of empathy, and possibly self-esteem. Music seems most likely to help develop empathy when it involves activities that encourage individual rather than synchronous group performance, yet within groups, tightly entrained with others’ performance, and emphasizing attention toward other people, prediction and mutual cooperation. This is not an aspect of music-making that has received attention in the literature on the personal and social benefits of joint music-making, although it is taught in some spheres of Western music-making, including jazz and chamber music. The present study suggests that it is also achievable in musically less-skilled interactive group improvisation. Players need to sequence and coordinate relatively independent playing, and hence to sensitively predict and respond to another person’s intentions and wishes. Note, however, that our musical turn-taking emulated conversational turn-taking: it was explicitly modeled on how conversational turns work, rather than on turn-taking and turn-taking signals typical of jazz and other ensemble playing.

We have hypothesized that music enhances empathy and self-esteem by different routes. Turn-taking may enhance empathy directly via enhanced interpersonal understanding and sensitivity, due to frequent temporally-coordinated reciprocal action, while joint synchrony may enhance personal well-being and hence prosociality directly, from which enhanced empathy develops indirectly later.

## Data Availability Statement

The raw data supporting the conclusions of this article will be made available by the authors, without undue reservation.

## Ethics Statement

The studies involving human participants were reviewed and approved by Ethics Committee of the School of Humanities and Social Sciences, University of Cambridge. Written informed consent to participate in this study was provided by the participants’ legal guardian/next of kin, and by the young participants themselves.

## Author Contributions

SH developed the study concept, negotiated with institutions, designed the experiments and many of the games, conducted the statistical analyses, lead the linguistic activities in each session, and had primary responsibility for writing the manuscript. CF contributed to design conceptualization and negotiation with institutions, session design and the content of musical activities, led session delivery, and contributed to the manuscript. Both authors worked together to assess the games’ validity and feasibility before use, and their success and any improvements immediately after each session.

## Conflict of Interest

The authors declare that the research was conducted in the absence of any commercial or financial relationships that could be construed as a potential conflict of interest.

## Publisher’s Note

All claims expressed in this article are solely those of the authors and do not necessarily represent those of their affiliated organizations, or those of the publisher, the editors and the reviewers. Any product that may be evaluated in this article, or claim that may be made by its manufacturer, is not guaranteed or endorsed by the publisher.

## Footnotes

1
https://psychology-tools.com/test/toronto-empathy-questionnaire

2
https://www.annarbor.co.uk/index.php?main_page=index&cPath=248_238

3
https://www.autismresearchcentre.com/tests/eyes-test-child/

4
https://www.youtube.com/watch?v=o10drRI3VQ0

5
http://excathedra.co.uk/education-participation/
