# Supplementary material for: Influence of Turn-Taking in Musical and Spoken Activities on Empathy and Self-Esteem of Socially Vulnerable Young Teenagers
Source: Front Psychol. 2022 Feb 7;12:801574. doi: 10.3389/fpsyg.2021.801574 (PMC8859432; doi:10.3389/fpsyg.2021.801574)
Supplement: Supplementary file 1 [file Data_Sheet_1.pdf]

Supplementary Material

Information about the psychometric tests and their use

1 TEQ

The TEQ comprises 16 statements to each of which the testee responds on a 5-point scale labelled Never, Rarely, Sometimes, Often, Always. Unlike some empathy measures, correlations are high between the TEQ and the RMIE (adult version), and the TEQ and several other empathy measures (Spreng, *et al.*, 2009).

2 RMIE

Each item in the Reading the Mind in the Eyes Test (children's version, since renamed the Eyes Test, Baron-Cohen, *et al.*, 2001) shows a photograph of a person's eye region, together with a set of four words or phrases, from which the participant chooses the word that best describes what that person is feeling. The RMIE is said to test empathetic accuracy rather than more cognitively-mediated responses or socially-acceptable responses that are a risk of self-report measures.

3 CFSEI-3

CFSEI-3 (Battle, 2002) comes in two forms, a 64-item version for 9-12 year olds, which yields scores for 4 subscales and a global self-esteem quotient, and a 67-item version for 13-18 year olds, which yields 5 subscales and a global quotient. Children took the appropriate one for their age. Children whose 13th birthday fell within the study period took the 12-year-old version if they were mainly 12 during the study period, and the 13-year-old version otherwise, so that each person took the same version before and after the sessions. Each form was completed by 11 children. Five Turntakers (3 F) and six Synchrony children (3 F) were 12 years old; six Turntakers (2 F) and five Synchrony children (3 F) were 13 or 14.

Baron-Cohen, S., Wheelwright, S., Spong, A., Scahill, V., and Lawson, J. (2001). Are intuitive physics and intuitive psychology independent? A test with children with Asperger Syndrome. *Journal of Developmental and Learning Disorders* 5**,** 47-78.

Battle, J. (2002). *Culture-Free Self-Esteem Inventories Examiner's Manual.* Austin, TX: pro-ed Inc.

Spreng, R.N., McKinnon, M.C., Mar, R.A., and Levine, B. (2009). The Toronto empathy questionnaire: Scale development and initial validation of a factor-analytic solution to multiple empathy measures. *Journal of Personality Assessment* 91(1)**,** 62-71. doi: 10.1080/00223890802484381.
