# Supplementary material for: Influence of Turn-Taking in Musical and Spoken Activities on Empathy and Self-Esteem of Socially Vulnerable Young Teenagers
Source: Front Psychol. 2022 Feb 7;12:801574. doi: 10.3389/fpsyg.2021.801574 (PMC8859432; doi:10.3389/fpsyg.2021.801574)
Supplement: Supplementary file 2 [file Table_1.pdf]

frontiers

Supplementary Material

Activities

Table S1 is discussed in the main text, in the section entitled "Activities", especially its subsection "Detailed Differences".

Plans for individual sessions 1-4 are available from the first author.

Table S1. Activities from Sessions 5 and 6 for the Turn-taking (left column) and Synchrony (right column) Groups. Sessions 5 and 6 were similar, since session 6 introduced no new material and rather focused on consolidating skills. In the sessions themselves, music and language activities roughly alternated. But here, musical activities are placed first, then language activities, so that the range covered by each is clearer. Activities with the same item number (e.g. 1T and 1S), are intended to be equivalent between the two groups except for the Turn-taking vs Synchrony experimental manipulation.

**Turntaking Group**	**Synchrony Group**
**1T) Djembe drumming: call and response (6-7 minutes)**<br><br>**Focus: encourage subtle, well-timed nods and looks and shared sense of pulse at turn transitions**<br><br>i. Facilitator plays a 2-bar rhythm on the drum (the call). The group imitates the rhythm together (response). Each participant then takes the role of leader, initiating a new 2-bar rhythm, which the rest of the group imitate together.<br><br>ii. A participant plays a 2-bar rhythm before passing it on to any other member of the group, indicating who to pass it to by looking and a nod of the head. The group aims to keep a shared pulse going throughout every person's turn.<br><br>iii. All participants drum a rhythm repeatedly together. Facilitator sings a 2-bar melody, which the group imitates together in response (call and response mode). Leadership is passed round the group as in i.	**1S) Djembe drumming (6-7 minutes)**<br><br>**Focus: encourage shared pulse; leading; changing and following dynamics; tidy starts and ends**<br><br>i. Facilitator plays a repeating 2-bar rhythm, which the participants play in synchrony with the facilitator.<br><br>ii. Each participant takes it in turn to act as 'leader' by improvising a new rhythm and indicating by facial and body movements when the group should start and stop playing with her, so that tight synchrony is maintained throughout.<br><br>iii. All participants drum the same rhythm repeatedly together. The group sing a familiar song in synchrony whilst drumming.
(continued)	(continued)

**2T) Balaphon blues (approx. 10 minutes)**<br><br>**Focus: learn blues rhythmic style, melodic improvisation, timing and prediction of turn transitions**<br><br>Facilitator teaches a 16-bar melody on the balaphons in a blues style, with piano accompaniment. This forms the 'chorus' of the piece and all group members play it in synchrony.<br><br>i. In the chorus, participants play in synchrony and shout 'hey' between phrases on the beat, with a raised arm and fist if wished. Leadership in setting the pulse etc may be passed around the group.<br><br>ii. In a 16-bar verse, two participants take turns to improvise 4-bar phrases with fluent turn transitions (2 turns each), while the other participants listen.<br><br>iii. The chorus-verse-chorus-verse structure continues until everyone has solo'd in 2 verses, totalling 4 turns in 16 bars for each player.<br><br>The participants are encouraged to maintain a shared basic pulse throughout the piece, especially at turn transitions.	**2S) Balaphon blues (approx. 10 minutes)**<br><br>**Focus: learn blues rhythmic style, melodic improvisation**<br><br>Facilitator teaches a 16-bar melody on the balaphons in a blues style, with piano accompaniment. This forms the 'chorus' of the piece and all group members play it in synchrony.<br><br>i. In the chorus, participants play in synchrony. Leadership in setting the pulse etc may be passed around the group.<br><br>ii. In the 16-bar verses, each participant improvises a melody in the blues style on their own, while the other participants listen.<br><br>iii. The chorus-verse-chorus-verse structure continues until everyone has had one turn improvising a solo verse.<br><br>The participants are encouraged to maintain a shared basic pulse throughout the piece.
**3T) Instrumental improvisation (approx. 10 minutes)**<br><br>**Focus: invent a phrase with a clear structure, turn-transition prediction and timing, shared rests**<br><br>Participants each invent their own one-bar (4 beat) rhythmic phrase on an instrument of their choice (balaphon, tambourine, djembe).<br><br>i. Participants play their phrase once each before passing to the next participant in the group (the 'receiver'), who enters in tempo.<br><br>ii. As for i, but each receiver waits for 1 beat before beginning their phrase. This is repeated except with each receiver waiting for 2 and then for 3 beats before starting their turn.	**3S) Instrumental improvisation (approx. 10 minutes)**<br><br>**Focus: learn a phrase with a clear structure, play sensitively in synchrony, precisely time shared rests**<br><br>Participants learn a one-bar (4 beat) rhythmic phrase, which incorporates a one-beat rest, on an instrument of their choice (balaphon, tambourine, djembe).<br><br>i. Participants play the phrase repeatedly in synchrony<br><br>ii. Each participant has one period as 'soloist'. The soloist invents a melody on the balaphon while the rest of the group play the repeated one-bar phrase in synchrony. Soloist's dynamics are responded to so that the solo can be heard.

iii. Participants play their phrase once, twice or three times before passing the turn to any chosen receiver, who begins the phrase without skipping a beat. Thus turn endings and/or the receiver are relatively unpredictable until just at the end of a turn. Receivers predict when to enter by attending closely to the current player's cues (beat slows, dynamic changes, body language, eye contact etc).	iii. The facilitator conducts the group and soloist in order to help the group come to the end of their improvisation and to finish together.
**4T) Group rap and body percussion (approx. 5 minutes)**<br><br>**Focus: contrast naturally-spoken and rapped rhythms, body movements synchronized with accented syllables**<br><br>Facilitator teaches participants a rap (e.g. Fresh Prince of Bel Air) or poem. Participants practice the difference between rapping and saying the words in a natural conversational style.<br><br>i. Participants chant one line each, then pass it on. Meanwhile, other participants tap or clap along on accented syllables.<br><br>ii. When spoken/rapped rhythms are secure, participants use a more complex, taught sequence of body percussion, passing that in turns as well,	**4S) Group rap and body percussion (10 minutes)**<br><br>**Focus: contrast naturally-spoken and rapped rhythms, body movements synchronized with accented syllables**<br><br>Facilitator teaches participants a rap (e.g. Fresh Prince of Bel Air), poem or limerick. Participants practice the difference between rapping and saying the words in a natural conversational style. Limericks allowed discussion of 3/4 rhythm whereas most other activities used 4/4.<br><br>i. Participants chant all lines of the rap/poem in synchrony. All participants clap together along with the beat or tap on a drum once the beat and words are secure.<br><br>ii. When spoken/rapped rhythms are secure, participants use a more complex, taught sequence of body percussion, and may stamp to exaggerate the pulse.<br><br>iii. Participants performed solo.<br><br>iv. Music (including piano) was added when the words and rhythm were secure to produce a good energy.
**5T) Asking directions (approx.10 minutes)**<br><br>**Focus: number of syllables and relative durations, to produce natural speech rhythms, pitch contours, well-timed interjections,**<br><br>Facilitator writes on a flip chart directions from current room to a familiar landmark, here, the school Reception.<br><br>*Go through the folding door; turn right; and through the 2 doors; along the Inclusion Courtyard; left through the gate; diagonally across, past the sign post; through the double doors at the far corner; Reception's on your left*.<br><br>i. Everyone taps or claps the rhythm in synchrony, speaking too if able to, as if giving directions.	**5S) Asking directions**<br><br>There was no exact Synchrony equivalent to activities like asking directions. Instead, the same amount of time was devoted to rap and/or other activities (as in 4S above) that involved language, including an emphasis on body movement (clapping, stamping, swaying) in time with the rhythm, and contrasting the differences between rapped and naturally-spoken rhythms and intonational ('melodic' pitch) contours.

<table>
<tr>
<td>ii. While others listen, one person says ‘Excuse me, where’s Reception?’, and a second person replies, saying each phrase as naturally as possible. At the end of each phrase, person 1 interjects e.g. with ‘yes’,’ uh-huh’ ‘ok’ as wished, while not interrupting person 2’s flow, and thanks the speaker at the end.

iii. Roles are reversed.

iv. Other pairs repeat ii and iii, sometimes the whole passage, sometimes passing single phrases and interjections around the group, while maintaining overall rhythm and flow; everyone else listens. (If time is short, the entire listening group interjects.) Normal speech rate and flow are encouraged.

v. If time, talker 2 introduces an ‘error’ which talker 1 corrects, to practice changing prosody e.g. ‘turn left?’ ‘No, turn RIGHT.’</td>
<td></td>
</tr>
<tr>
<td>6T) Balaphon duets: conversations (approx. 10 minutes)

Focus: natural speech rhythms, listener predicts pauses of speaker, well-timed interjections

Participants have a balaphon each and work in pairs. One participant in each pair is the ‘speaker’ while their partner is the ‘responder’.

i. The speaker plays phrases on the balaphon with natural speech rhythms. Participants were given example phrases e.g. “What did you have for breakfast?” They are encouraged to use rhythms of natural speech rather than to adhere to a musical pulse. They may get faster or slower in tempo, and can produce longer ‘utterances’ than the above example, but they should mimic on the balaphon the rhythm and approximate pitch contour of specific utterances.

ii. The responder interjects with short 1-3 note phrases at any slight pauses between the speaker’s phrases, and can give longer responses when the first player pauses for long enough.

iii. The participants exchange roles.</td>
<td>6S) Balaphon duets: speech rhythms (approx. 5 mins)

Focus: 1-to-1 coordinated interaction with independent roles, starting and stopping together, pulse synchrony, sensitivity to speaker/follower roles

Participants have a balaphon each and work in pairs. One participant is designated ‘speaker’ and the other ‘follower’.

i. The speaker plays phrases on the balaphon with natural speech rhythms. At the same time, and using any instrument(s), the follower plays beats in time with the speaker’s ‘accented syllables’. Participants were given example phrases e.g. “I often go swimming on Saturdays”. They are encouraged to use rhythms of natural speech rather than to adhere to a musical pulse.

ii. The participants exchange roles.</td>
</tr>
<tr>
<td>(continued)</td>
<td>(continued)</td>
</tr>
</table>

7T) Song performance (approx. 15 minutes)	7S) Song performance (approx. 15 minutes)
**Focus: consolidate turn transition skills in the context of an enjoyable song 'performance'**	**Focus: consolidate synchrony skills in the context of an enjoyable song 'performance'**
Using the song 'Rather Be' by Clean Bandit, each section of the song is treated with an emphasis on turn passing and maintaining a constant pulse between turns.	Using the song 'Rather Be' by Clean Bandit, each section of the song is treated with an emphasis on synchrony, memory of complex body percussion sequence and maintaining a constant pulse.
i. INTRODUCTION: The opening motif of Rather Be is performed as a rhythmic sequence using body percussion. Each participant claps one bar of the rhythm, before passing to the next person, together forming the full rhythmic sequence.	i. INTRODUCTION: The opening motif of Rather Be is performed as a rhythmic sequence using body percussion. All participants clap the full sequence in synchrony.
ii. VERSE 1: The rhythmic percussive sequence continues to be passed around the participants while everyone sings the first verse together in unison.	ii. VERSE 1: The rhythmic percussive sequence continues to be clapped in synchrony while they sing the first verse together in unison
iii. MID-SECTION: Split into 6 lines of text, each rapped by a different member of the group in sequence. Between each line, all other members of the group shout an interjection e.g. 'uh-huh' in synchrony, if wished with raised arm and fist.	iii. MID-SECTION: All participants rap the mid-section together in synchrony.
iv. CLAPPING SEQUENCE: The rhythmic sequence is clapped and passed round the group members in sequence.	iv. CLAPPING SEQUENCE: All participants clap the rhythmic sequence in synchrony.
v. CHORUS: All participants sing in unison, clap and beat drums in time together.	v. CHORUS: All participants sing in unison, clap and beat drums in time together.
vi. ENDING: The rhythmic sequence is clapped and passed round the group members in sequence	vi. ENDING: All participants clap the rhythmic sequence in synchrony.
